# Genomic Profiling of *Mycobacterium tuberculosis* Strains, Myanmar

**DOI:** 10.3201/eid2711.210726

**Published:** 2021-11

**Authors:** Htin Lin Aung, Wint Wint Nyunt, Yang Fong, Patrick J. Biggs, Richard C. Winkworth, Peter J. Lockhart, Tsin Wen Yeo, Philip C. Hill, Gregory M. Cook, Si Thu Aung

**Affiliations:** University of Otago, Dunedin, New Zealand (H.L. Aung, P.C. Hill, G.M. Cook);; Ministry of Health and Sports, Yangon, Myanmar (W.W. Nyunt);; Massey University, Palmerston North, New Zealand (Y. Fong, P.J. Biggs, R.C. Winkworth, P.J. Lockhart);; Nanyang Technological University, Singapore (T.W. Yeo);; Ministry of Health and Sports, Naypyitaw, Myanmar (S.T. Aung)

**Keywords:** Mycobacterium tuberculosis, bacteria, strains, tuberculosis and other mycobacteria, tuberculosis, respiratory infections, genomic profiling, antimicrobial resistance, expanded drug resistance testing, new oral regimens, Myanmar

## Abstract

Multidrug resistance is a major threat to global elimination of tuberculosis (TB). We performed phenotypic drug-susceptibility testing and whole-genome sequencing for 309 isolates from 342 consecutive patients who were given a diagnosis of TB in Yangon, Myanmar, during July 2016‒June 2018. We identified isolates by using the GeneXpert platform to evaluate drug-resistance profiles. A total of 191 (62%) of 309 isolates had rifampin resistance; 168 (88%) of these rifampin-resistant isolates were not genomically related, indicating the repeated emergence of resistance in the population, rather than extensive local transmission. We did not detect resistance mutations to new oral drugs, including bedaquiline and pretomanid. The current GeneXpert MTB/RIF system needs to be modified by using the newly launched Xpert MTB/XDR cartridge or line-probe assay. Introducing new oral drugs to replace those currently used in treatment regimens for multidrug-resistant TB will also be useful for treating TB in Myanmar.

Tuberculosis (TB) is the infectious disease that causes the most deaths worldwide (≈5,000/day) (*1*). Of major concern is the increasing prevalence of drug resistance worldwide (*1*). There are different forms of TB drug resistance: pre‒multidrug-resistant TB (pre‒MDR TB, resistant to 1 of 2 first-line drugs: isoniazid or rifampin); multidrug-resistant TB (MDR TB, resistant to 2 first-line drugs: isoniazid and rifampin); pre‒extensively drug-resistant (pre-XDR, resistant to either fluoroquinolones or injectable drugs in addition to MDR); and extensively drug-resistant TB (XDR TB, resistant to fluoroquinolones and injectable drugs in addition to MDR) (*1*). An estimated 0.5 million cases of MDR TB were reported in patients worldwide during 2018, but only one third had access to effective treatment, resulting in 56% of patients being successfully treated (*1*). In addition, an estimated 6% of diagnosed case of MDR TB cases are actually cases of XDR TB (*1*).

Myanmar is recognized by the World Health Organization (WHO) as having high burdens of TB (338 cases/100,000 population), MDR TB (21 cases/100,000 population), and co-infections of TB and HIV (29 cases/100,000 population) (*1*). A nationwide drug-resistant TB survey was conducted during 2012–2013 by the Myanmar National Tuberculosis Programme (NTP) to identify the drug susceptibility profile for first-line drugs (phenotypic drug susceptibility testing for second-line drugs was established during 2016) (*2*). This survey identified MDR TB among 5% of new cases and 27.1% of previously treated cases, and the Yangon region was identified as a hotspot for drug-resistant TB (*3*). Having an estimated population of 8 million persons, Yangon is the most populous city in Myanmar. All patients with suspected pulmonary TB are referred to a TB diagnostic center run by the NTP for testing by using the GeneXpert platform (https://www.cepheid.com). Routine, phenotypic drug-susceptibility testing (DST) of first-line or second-line drugs is rarely performed for new patients, and currently testing is based solely on the Xpert MTB/RIF (*M. tuberculosis*/rifampin) assay. Therefore, clinical decisions reflect the detection of rifampin resistance and national therapeutic guidelines on the basis of WHO recommendations.

Technological advances in next-generation, whole-genome sequencing (WGS) and downstream bioinformatic analyses now enable comprehensive detection of drug resistance and provide an alternative to existing approaches (*3*–*5*). Such sequence-based, drug-resistant profiles have high concordance with phenotypic DST (*3*,*4*). In addition, phylogenetic analyses of sequence data can be used to identify transmission patterns in the absence of epidemiologic data, which is often lacking in high-burden settings such as Myanmar (*3*,*6*). We combined clinical, genomic, and phenotypic drug-resistance data to provide insights into drug resistance and transmission patterns in Yangon. In this study, we used WGS analyses of 309 *M. tuberculosis* isolates to determine how the increasing burden of MDR TB has been driven in Yangon.

## Methods

### Study Design and Participants

This population-based, cross-sectional study included consenting participants >15 years of age who had GeneXpert-confirmed positive pulmonary TB at 3 major NTP TB diagnostic centers (Aung San, Latha, and North Oakkalapa) in Yangon during July 2016‒June 2018. We aimed to recruit 250 patients consecutively given a diagnosis of infection with rifampin-resistant (RR) *M. tuberculosis* and 200 patients infected with rifampin-susceptible (RS) *M. tuberculosis*. Recruitment numbers at each facility reflected the relative numbers of patients given a diagnosis during the previous year. Patients were eligible to be included in the study if they had lived in Yangon at the time of registration, had a TB-positive confirmation by GeneXpert, and provided written informed consent. Patients were excluded if their residential address was outside Yangon at the time of registration or they did not provide informed consent. We obtained a brief clinical report for each patient (basic demographics, residential address, history of TB treatment, HIV status, and random blood glucose testing results for diabetes mellitus). The Institutional Review Boards of the Department of Medical Research, Ministry of Health and Sports of Myanmar, and the Human Health Ethics Review Committee of the University of Otago (Dunedin, New Zealand) approved this study.

### Laboratory Procedures

We collected all clinical sputum samples at the time of diagnosis and before commencement of treatment. We sent samples to the National Tuberculosis Reference Laboratory in Yangon for DST. Testing for resistance to isoniazid, rifampin, ethambutol, streptomycin, para-aminosalicylic acid, ethionamide, D-cycloserine, fluoroquinolones (ofloxacin, levofloxacin, capreomycin), and aminoglycosides (amikacin, kanamycin) was performed by using the proportion method on Löwenstein‒Jensen medium (https://apps.who.int/iris/bitstream/handle/10665/83807/WHO_CDS_TB_2001.288_eng.pdf). We determined resistance to new and repurposed drugs (i.e., pyrazinamide, bedaquiline, pretomanid, delamanid, linezolid, and clofazimine) on the basis of genomic markers known to be associated with resistance (*5*,*7*,*8*). Clinicians were provided with the WGS and accompanying phenotypic DST data as soon as it was available, and clinical decisions were made entirely at their discretion.

We extracted genomic DNA from cultures of single sputum specimens by using MoBio Microbial DNA Isolation Kits (https://www.qiagen.com) and sequenced DNA by using Illumina MiSeq (https://www.illumina.com) as described (*9*,*10*). All sequencing data from this study were deposited into the National Center for Biotechnology Information Sequence Read Archive (https://www.ncbi.nlm.nih.gov/sra; accession no. PRJNA638161).

### Analysis

We performed genomic mapping by using Burrow-Wheeler Aligner-maximum exact matches (version 7.17-r1188; https://bio-bwa.sourceforge.net) and the *M. tuberculosis* reference genome H37Rv (GenBank accession no. NC_000962.3). Mapping used a custom *M. tuberculosis* masking browser extensible data file to exclude highly repetitive GC-rich conserved domains. We used SAMtools and BCFtools utilities version 1.9 to call single-nucleotide polymorphisms (SNPs) (*11*). *M. tuberculosis* TB-Profiler version 2.8.2 (https://github.com) was used to predict resistance to 17 drugs on the basis of genotyping of gene targets and classification to phylogenetic lineages by using SNP barcodes (*7*,*8*). Maximum-likelihood phylogenetic analyses were conducted by using RaxML, as implemented in the Gubbins pipeline version 2.3.4 (*12*). We used the online platform iTOL version 5.5 for annotation and management of phylogenetic trees (*13*). Isolates were considered closely related (genomically linked) if the pairwise distance between them was <12 SNPs (*5*). Statistical analyses were performed by using GraphPad Prism version 8.0 (https://www.graphpad.com) and the χ^2^ test. A p value <0.05 was considered statistically significant.

## Results

Over the recruitment period, 342 patients (194 with RR and 148 with RS *M. tuberculosis*) participated in the study; 33 case-patients were excluded because of laboratory contamination or failed sputum culture and DNA extraction. Of the final 309 GeneXpert-positive included participants, 200 (65%) were male (Table 1), 118 were RS and 191 were RR, and all had phenotypic DST successfully completed. RR was strongly associated with a history of TB treatment (p<0.0001) (Table 1).

**Table 1:** Characteristics of patients who were infected with rifampin-susceptible and rifampin-resistant *Mycobacterium tuberculosis* strains that were identified by using Xpert MTB/RIF assay, Myanmar*

Characteristic	Resistant, n = 191	Susceptible, n = 118	p value
Sex			
M	120	80	0.39
F	71	38	
Treatment history			
Retreatment	108	31	<0.0001
New	83	87	
District			
North	64	74	<0.0001
South	7	3	
East	75	31	
West	45	10	
Age, y			
10–19	7	4	0.90
20–39	102	59	
40–59	67	44	
>60	15	11	
HIV			
Positive	6	1	0.28
Negative	200	102	
Random blood glucose, mg/dL			
>200	13	8	0.64
<200	193	95	
Laboratory testing			
Lineage 2	164	37	<0.0001
Other	27	81	

*Xpert MTB/RIF (*M. tuberculosis*/rifampin), Cepheid (https://www.cepheid.com).

**Figure 1:**
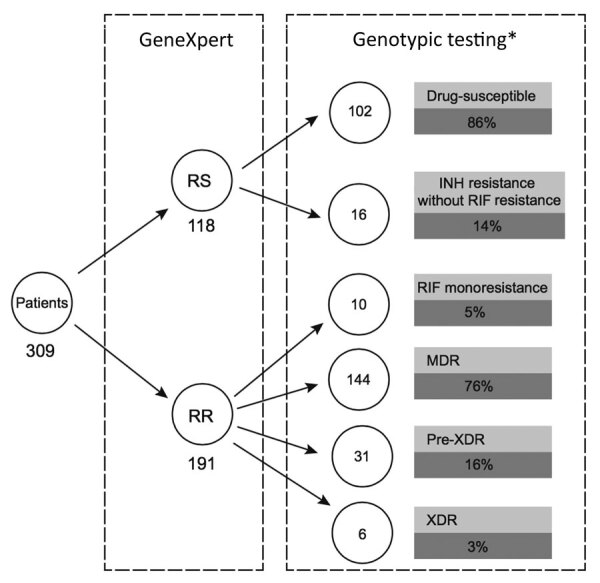
Genomic profiling of *Mycobacterium tuberculosis* strains, Myanmar, comparing discriminatory power offered by GeneXpert (Cepheid, https://www.cepheid.com) and additional genotypic testing, such as line-probe assay or whole-genome sequencing. RIF resistance and sensitivity were determined by using the Xpert MTB/RIF assay (Cepheid). INH, isoniazid; MDR, multidrug resistant; RIF, rifampin; RR, rifampin resistant; RS rifampin sensitive; XDR, extensively drug resistant. *Resistance profile confirmed by phenotypic testing.

We compared the results of the GeneXpert, phenotypic DST, and genomic analyses to further evaluate drug resistance (Figure 1). Of 118 cases diagnosed as RS by using GeneXpert, 16 (14%) were identified as isoniazid resistant on the basis of genomic analyses (Figure 1); resistance was conferred either by a mutation in the *katG* gene (S315T; 12 [75%] of 16) or in the promoter region of the *inhA* gene (c-15t; 4 [25%] of 16) (Table 2; Figure 1). All 16 cases were phenotypically confirmed as isoniazid resistant (Table 2).

**Table 2:** Comparison of phenotypic drug susceptibility testing and genomic resistance mutation results for *Mycobacterium tuberculosis* strains, Myanmar*

Drug	Performance of genome-based† drug resistance profile prediction with respect to phenotypic drug-susceptibility testing						
	Mutation			No mutation		Sensitivity, %	Specificity, %
	Sensitive	Resistant		Sensitive	Resistant		
Isonizaid	0	196		113	0	100.0	100.0
Rifampin	0	191		118	0	100.0	100.0
Ethambutol	34	80		155	40	70.2	79.5
Streptomycin	26	140		80	63	84.3	55.9
Ofloxacin/levofloxacin/capreomycin	13	31		263	2	70.4	99.2
Amikacin/kanamycin	0	6		303	0	100.0	100.0
Para-aminosalicylic acid	15	0		294	0	NA	100.0
Ethionamide	4	28		274	3	87.5	98.9
D-cycloserine	0	0		309	0	NA	100.0

*NA, not applicable. †Whole-genome sequencing‒based prediction.

**Figure 2:**
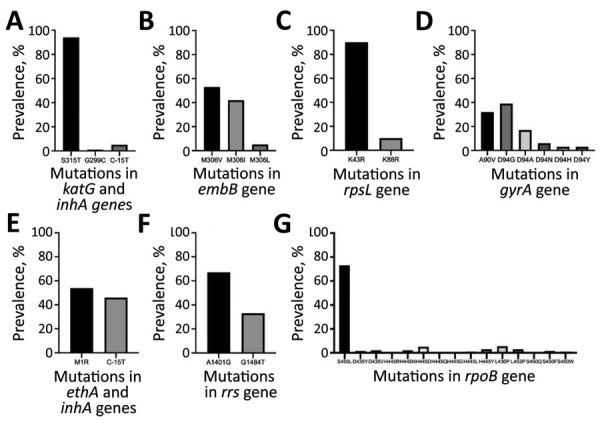
Prevalence of resistance-conferring mutations in genes of phenotypically resistant isolates of *Mycobacterium tuberculosis* strains, Myanmar. A) *katG* and *inhA*; B) *embB*; C) *rpsL*; D) *gyrA*; E) *ethA* and *inhA*; F) *rrs*; G) *rpoB*.

All 191 RR isolates identified by GeneXpert were phenotypically resistant; the S450L mutation in the *rpoB* gene was the dominant mutation (137 [72%] of 191 (Table 2; Figure 2). WGS further identified that 10 (5%) were only rifampin resistant (pre-MDR), 144 (75%) were MDR, 31 (16%) were pre-XDR, and 6 (3%) were XDR; results were confirmed by phenotypic DST (Figure 1). All pre-XDR isolates harbored mutations in the *gyrA* gene, and D94G was most prevalent (12 [39%] of 31), followed by A90V (8 [26%] of 31) (Table 2; Figure 2). Resistance to aminoglycoside injectable drugs (XDR) was predominantly associated with *rrs* A1401G (4 [6 [67%] of 6) and G1484T (2 [33%] of 6) mutations. Mutations in the *embB* gene (M306V; 42 [53%] of 80), the M306I mutant (34 [43%] of 80), and mutations in the *rpsl* gene (K43R; 126 [90%] of 140) were present in all ethambutol-resistant and streptomycin-resistant isolates, resulting in a sensitivity of 70.2% and a specificity of 79.5% for ethambutol and a sensitivity of 84.3% and a specificity of 55.9% for streptomycin (Table 2; Figure 2). Known mutations conferring resistance to new and repurposed drugs, such as bedaquiline, delamanid, pretomanid, linezolid and clofizamine, were not identified by WGS in the 207 drug-resistant isolates (26 pre-MDR, 144 MDR, 31 pre-XDR, 6 XDR).

**Figure 3:**
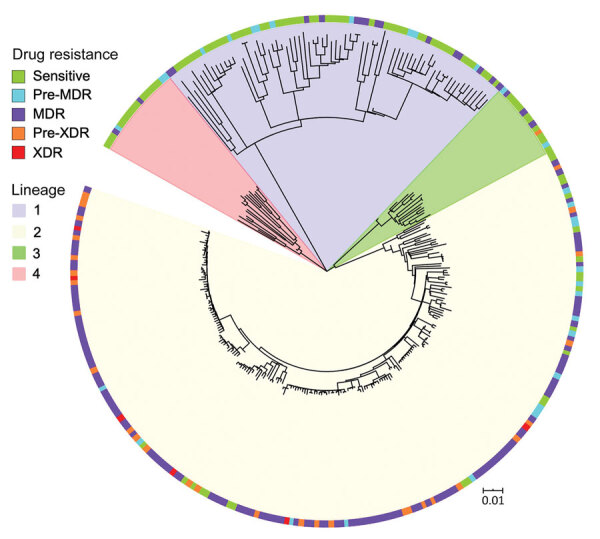
Maximum-likelihood tree based on whole-genome analysis of 309 *Mycobacterium tuberculosis* strains from Myanmar. Lineages and drug resistance status of isolates are shown. MDR indicates multidrug-resistant to 2 first-line drugs (isoniazid and rifampin); pre-MDR, resistant to 1 of 2 first-line drugs (isoniazid or rifampicin); pre-XDR, resistant to fluoroquinolones or injectable drugs in addition to MDR; XDR, resistant to fluoroquinolones and injectable drugs, in addition to MDR. Scale bar indicates nucleotide substitutions per site. MDR, multidrug resistant; XDR, extensively drug resistant.

**Figure 4:**
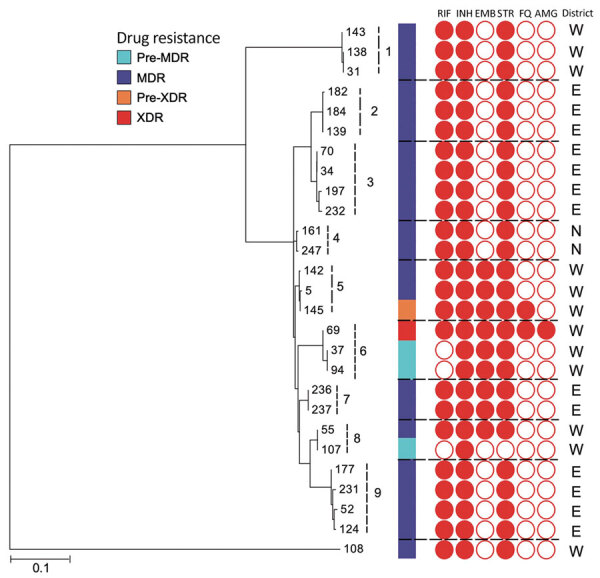
Maximum-likelihood tree of *Mycobacterium tuberculosis* strains, Myanmar, within 9 clusters and their drug resistance profiles. Dotted lines indicate boundaries of individual clusters. An outgroup (#108) differs by >100 single-nucleotide polymorphisms from the strains within 9 clusters. N, E, and W indicate the North, East, and West Districts of Yangon, respectively. MDR, resistant to 2 first-line drugs (isoniazid and rifampin); pre-MDR, resistant to 1 of 2 first-line drugs (isoniazid or rifampicin); pre-XDR, resistant to fluoroquinolones or injectable drugs in addition to MDR; XDR, resistant to fluoroquinolones and injectable drugs, in addition to MDR. AMG, aminoglycosides; ETH, ethambutol; FQ, fluoroquinolones; INH, isoniazid; RIF, rifampin; STR, streptomycin. Scale bar indicates nucleotide substitutions per site.

Using specific SNP barcodes, we classified the *M. tuberculosis* isolates as either lineage 1 (73, 24%), lineage 2 (201, 65%), lineage 3 (16, 5%) or lineage 4 (19, 6%) (Figure 3; Appendix Table 1). Most isolates were identified as belonging to sublineage 2.2.1, Beijing strain (Appendix Table 1). Isolates linked to TB lineage 2 were more commonly drug resistant than those belonging to other lineages (175 [85%] of 207 vs. 32 [15%] of 207; p<0.0001). In contrast, the other lineages were more commonly associated with drug susceptibility (76 [75%] of 102 vs. 26 [25%] of 102; p<0.001) (Table 1; Appendix Table 2). Drug-resistant isolates were also more commonly found in the East and West districts of Yangon (124 [60%] of 207; p<0.0001) (Table 1; Appendix Table 2) and to be associated with patients who had previously received treatment (112 [54%] of 207; p<0.0001) (Appendix Table 2). A total of 181 [87%] of 207 isolates were genomically unlinked on the basis of a standard pairwise distance threshold. The remaining 26 (13%) of 207 drug-resistant isolates formed 9 potential transmission chains (Figure 4). 

Cases within most of these groups were located within the same districts (Figure 4), and each group contained a combination of new and previously treated TB patients. In 6 groups, all isolates had the same resistance profile; the remaining 3 (i.e., groups 5, 6, and 8) groups, had different resistance profiles. In group 6, an XDR isolate appears to have developed from an isoniazid-resistant (pre-MDR) isolate (Figure 4).

## Discussion

This WGS study from Myanmar provides new insights into the landscape of drug-resistant TB in the country’s largest city. A large proportion of isolates with high-level drug resistance, including pre-XDR and XDR, were identified. However, there was no resistance to new and repurposed drugs, such as bedaquiline, pretomanid, delamanid, and linezolid. Most drug-resistant cases were associated with previous treatment, and few were clearly associated with community transmission. These findings suggest an additional diagnostic tool, such as the Xpert MTB/XDR cartridge or line-probe assay (LPA), in addition to Xpert MTB/RIF, and new oral regimens, including bedaquiline and pretomanind, are needed for effective surveillance and treatment/management of MDR TB in Mynamar. Further studies are also required to investigate apparent cases of independent emergence and community transmission of MDR TB in Yangon.

Consistent with previous reports on lineage 2 from neighboring countries, this study identified a strong association between lineage 2 *M. tuberculosis* and drug resistance (*14*–*17*). There was strong agreement between WGS (presence of resistance-conferring mutations) and phenotypic DST to isoniazid and rifampin in this study (*18*). These findings indicate quality assurance in the TB laboratory diagnostic service provided by the Myanmar National Tuberculosis Reference Laboratory.

The Xpert MTB/RIF assay has been effective in the simultaneous detection of TB and resistance to rifampin. Because it can provide a diagnosis for a patient within 2 hours, GeneXpert is critical in TB control in high-burden settings. One of the limitations of current cartridges for Xpert is that resistance to isoniazid is assumed when rifampin resistance is detected. This approach captures a large portion of drug-resistant TB cases during diagnosis. However, for a few case-patients, which includes patients who have isoniazid resistance without concurrent rifampin resistance (14% in this study and 9.4% in the recently reported multicountry study [*19*]), treatment with a first-line regimen can contribute to the emergence of further drug resistance.

We previously reported that a patient with undiagnosed isoniazid resistance without concurrent rifampin resistance received a first-line treatment regimen that resulted in development of MDR TB (*20*). This finding highlights the limitations and real-world consequences of basing treatment decisions solely on results of the GeneXpert MTB/RIF system in a high-burden setting, where hundreds of cases are reported daily. This limitation is a serious impediment to controlling the spread of more extensive drug resistance (*21*). For example, although Xpert MTB/RIF can correctly diagnose RR MDR cases, it cannot detect pre-XDR and XDR cases. As identified in this study, 20% of rifampin-resistant cases identified by GeneXpert were pre-XDR (17%) and XDR (3%) cases, suggesting that ≈1 of 5 patients received limited treatment on the basis of treatment guidelines at the time of the study.

Most case-patients (including pre-XDR and XDR patients) in this study had drug-resistant isolates that were not closely related (genomically unlinked), which is suggestive of independent emergence of drug resistance because of limited diagnosis or treatment, as well as patient noncompliance. This finding is in contrast to previous studies from other high burden settings, such as China and South Africa, which showed a high proportion of drug-resistant cases that were genomically linked, suggesting community transmission (*15*,*21**–**23*).

Although it is possible that we simply did not have a high enough sampling fraction of all drug-resistant cases in the population under study, a high number of unclustered drug-resistant cases could be caused by differences in population density; the North, East, and West sections of Yangoon are in an urban industrial setting. These districts have a considerable factory-based workforce and thus draw in highly mobile migrant populations (internal migration), including members from neighboring states and regions, for employment (*24*). This finding enables a continuous flow of persons from outside Yangon, which could be independently introducing infections into the region. In addition, their status as migrants means they might have limited access to healthcare services, which is a barrier to rapid diagnosis and appropriate treatment for TB, underscoring the effect of migration on the TB burden in cities in Myanmar, particularly Yangon (*20*,*25*).

In addition to internal migration, cross-border migration has occurred in recent years, such as ≈6 million persons from fellow Greater Mekong Subregion (GMS) countries Cambodia, Laos, Thailand, and Vietnam. Therefore, the Myanmar NTP is collaborating with nongovernmental organizations and NTPs from other GMS countries to reduce the TB burden among Myanmar migrants. Further WGS studies outside Yangon and along these GMS borders are required to provide an insight into the transmission patterns of MDR TB in migrants. Coupling this collaboration with TB-related health education and increase access to care could ultimately reduce the TB burden among migrants.

Further studies are also required to clarify the limitations and roles of both public and private healthcare providers in current treatment pathways for TB in Yangon, which might be contributing to the high rates of MDR TB. In our study, WGS showed a chain of infection, leading to the progression of pre-MDR cases toward XDR and subsequent transmission events, highlighting the need for effective diagnosis. This finding has implications for public health policies and also shows the need for local data to drive effective intervention.

Our study has major implications for clinical practice in Myanmar. First, effective treatment for MDR TB cases requires identification of the high proportion of pre-XDR and XDR TB, which cannot be achieved by current Xpert MTB/RIF testing (*27*,*28*). The drug resistance‒conferring mutations reported in this study can be detected by first-line and second-line LPA, such as GenoType MTBDRplus and MTBDR*sl* (Hain Lifescience GmbH, https://www.hain-lifescience.de), or the recently launched Xpert MTB/XDR (*28*,*29*). These platforms can provide clinicians with an expanded drug-susceptibility report without the need for culturing and WGS. Recently, the Myanmar National Tuberculosis Programme diagnostic algorithm has been updated to extend first-line LPA for patients with a history of previous treatment. Second, several new or repurposed drugs (i.e., bedaquiline, delamanid linezolid, and pretomanid) are drugs already available in Myanmar. The apparent absence of preexisting mutations that confer resistance to these drugs justifies their introduction into treatment regimens for drug-resistant TB in Myanmar, as per WHO recommendations (*30*–*32*).

Our study has limitations that could lead to overestimation and underestimation of the true magnitude of the MDR epidemic and might not reflect the national situation. First, a large cohort of MDR TB, pre-XDR TB, and XDR TB cases was identified. The study region in Yangon is known to be a high-burden setting compared with other regions of Myanmar, accounting for ≈50% of all national cases (*26*). The 3 diagnostic centers in this study are also the major drug-resistant TB treatment centers in Yangon. Therefore, it is likely that the landscape of infections is not representative of all Myanmar. Another limitation is that the study timeframe and size make it unlikely that we captured a full spectrum of MDR TB strains in the population and, as noted earlier, we might have missed identification of some transmission links, thus overestimating the proportion of resistant isolates that are independent.

Our study has major implications for clinical practice in Myanmar. First, effective treatment for MDR TB cases requires identification of the high proportion of pre-XDR and XDR TB, which cannot be achieved by current Xpert MTB/RIF testing (*27*,*28*). The drug resistance‒conferring mutations reported in this study can be detected by first-line and second-line LPA, such as GenoType MTBDRplus and MTBDR*sl* (Hain Lifescience GmbH, https://www.hain-lifescience.de), or the recently launched Xpert MTB/XDR (*28*,*29*). These platforms can provide clinicians with an expanded drug-susceptibility report without the need for culturing and WGS. Recently, the Myanmar National Tuberculosis Programme diagnostic algorithm has been updated to extend first-line LPA for patients with a history of previous treatment. Second, several new or repurposed drugs (i.e., bedaquiline, delamanid linezolid, and pretomanid) are drugs already available in Myanmar. The apparent absence of preexisting mutations that confer resistance to these drugs justifies their introduction into treatment regimens for drug-resistant TB in Myanmar, as per WHO recommendations (*30*–*32*).

Our study is useful for public health officials in designing interventions for an evidence-based approach for early detection of cases (active case finding) with optimized diagnosis and treatment. Introducing additional diagnostic methods, such as routine LPA or Xpert MTB/XDR in tandem with Xpert MTB/RIF, and treatment regimens with new oral drugs would further assist in controlling and containing MDR TB in Myanmar. In addition, this study underscores the need for local data, rather than being based on general information from similar studies that have different healthcare delivery systems to drive public health policies for effective intervention.

Appendix Additional information on genomic profiling of *Mycobacterium tuberculosis* strains, Myanmar.
