# Supplementary material for: Genomic Profiling of Mycobacterium tuberculosis Strains, Myanmar
Source: Emerg Infect Dis. 2021 Nov;27(11):2847–55. doi: 10.3201/eid2711.210726 (PMC8544997; doi:10.3201/eid2711.210726)
Supplement: Appendix — Additional information on genomic profiling of Mycobacterium tuberculosis strains, Myanmar. [file 21-0726-Techapp-s1.pdf]

Article DOI: https://doi.org/10.3201/eid2711.210726

Genomic Profiling of *Mycobacterium tuberculosis* Strains, Myanmar

Appendix

Appendix Table 1. Frequency distribution of major and sublineages of *Mycobacterium tuberculosis* isolates, Myanmar

Major lineage	Sublineage	No. isolates	Total, no. (%)
Lineage 1	1.1.1	7	73 (24)
	1.1.2	21	
	1.1.3	31	
	1.2.1	10	
	1.2.2	4	
Lineage 2	2.2.1	195	201 (65)
	2.2.2	6	
Lineage 3	3	8	16 (5)
	3.1.2	8	
Lineage 4	4.1	8	19 (6)
	4.3	1	
	4.4	1	
	4.5	5	
	4.7	1	
	4.8	3	
Total			309 (100)

Appendix Table 2. Sociodemographic characteristics of patients in this study and phenotypic DST results, Myanmar*

		Drug resistance, n = 207				
Characteristic	Sensitive, n = 102	Pre-MDR, n = 26	MDR, n = 144	Pre-XDR, n = 31	XDR, n = 6	p value
Sex						
M	69	19	91	15	6	0.08
F	33	7	53	16	0	
Treatment history						
Retreatment	27	9	80	19	4	<0.0001
New	75	17	64	12	2	
District						
North	63	16	52	6	1	<0.0001
East	28	6	58	13	1	
South	2	2	5	0	1	
West	9	2	29	12	3	
Lineage						
Lineage 1	51	7	15	0	0	<0.0001
Lineage 2	26	15	124	30	6	
Lineage 3	11	1	3	1	0	
Lineage 4	14	3	2	0	0	
Genomically linked						
Clustered	8	3	21	1	1	
Unclustered	94	23	123	30	5	
Age, y						
10–19	3	1	5	1	1	0.7
20–39	52	12	73	20	4	
40–59	37	12	54	7	1	
>60	10	1	12	3	0	

*pre-MDR, pre–multidrug-resistant (resistant to 1 of 2 first-line drugs: isoniazid [INH] or rifampin [RIF]); MDE, multidrug-resistant (resistant to 2 first-line drugs: INZ and RIF); pre-XDR, pre-extensively drug-resistant (resistant to either fluoroquinolones or injectable drugs in addition to MDR); XDR, extensively drug-resistant (resistant to fluoroquinolones and injectable drugs in addition to MDR).
